# Effect of Cell Shape and Dimensionality on Spindle Orientation and Mitotic Timing

**DOI:** 10.1371/journal.pone.0066918

**Published:** 2013-06-18

**Authors:** Mirren Charnley, Fabian Anderegg, René Holtackers, Marcus Textor, Patrick Meraldi

**Affiliations:** 1 Laboratory for Surface Science and Technology, ETH Zurich, Zurich, Switzerland; 2 Institute of Biochemistry, ETH Zurich, Zurich, Switzerland; University of Birmingham, United Kingdom

## Abstract

The formation and orientation of the mitotic spindle is a critical feature of mitosis. The morphology of the cell and the spatial distribution and composition of the cells' adhesive microenvironment all contribute to dictate the position of the spindle. However, the impact of the dimensionality of the cells' microenvironment has rarely been studied. In this study we present the use of a microwell platform, where the internal surfaces of the individual wells are coated with fibronectin, enabling the three-dimensional presentation of adhesive ligands to single cells cultured within the microwells. This platform was used to assess the effect of dimensionality and cell shape in a controlled microenvironment. Single HeLa cells cultured in circular microwells exhibited greater tilting of the mitotic spindle, in comparison to cells cultured in square microwells. This correlated with an increase in the time required to align the chromosomes at the metaphase plate due to prolonged activation of the spindle checkpoint in an actin dependent process. The comparison to 2D square patterns revealed that the dimensionality of cell adhesions alone affected both mitotic timings and spindle orientation; in particular the role of actin varied according to the dimensionality of the cells' microenvironment. Together, our data revealed that cell shape and the dimensionality of the cells' adhesive environment impacted on both the orientation of the mitotic spindle and progression through mitosis.

## Introduction

The orientation of the mitotic spindle along a predetermined axis during mitosis plays an important role in cell fate and organ development [1]–[5]. Misorientation of the mitotic spindle has been implicated as a contributing factor in tumor development and polycystic kidney disease [6], [7]. Cell shape dictates the orientation of the mitotic spindle in many systems [8]–[12]. Cells orientate the mitotic spindle parallel to their long axis, resulting in cleavage along the shortest dimension of the cell [9], [11]. However, the orientation of the mitotic spindle is not controlled by cell shape alone. Théry et al. used patterned 2D substrates to demonstrate that anisotropy within the adhesive environment also guides the orientation of the mitotic spindle [13]. The arrangement and geometry of the cells' adhesive environment directs the localization of focal adhesions and associated stress fibers [14], [15]. Traction forces exerted on the focal adhesions culminate in the translation of the spatial distribution of the adhesive environment into a complementary cell traction force field [16], [17]. During mitosis the cell rounds up and the stress fibers within the cells disassemble [18] leaving the cell attached to the substrate via retraction fibers [13], [19], which subsequently direct spindle orientation [13], [20]. The spatial organization of these retraction fibers is determined by the spatial organization of traction forces and cortical cues within the cell during interphase [13], [21]. These cortical cues may be either intrinsic, such as asymmetrically distributed cortical factors [22], or extrinsic, such as cell–cell or cell-matrix adhesions [23], [24]. Anisotropy of the adhesive environment of the cell can alter the orientation of the mitotic spindle, independently of changes in global cell shape [13]. Conversely, surface anisotropy can alter the cell shape and alignment, and consequently the orientation of the mitotic spindle [25], [26]. Thus, the orientation of the mitotic spindle is controlled by cell shape and the distribution of the adhesive environment of the cell during interphase.

Currently, it is unclear how these changes in orientation impact on the progression of the cell through mitosis. The cell cycle, including mitosis, is rigorously controlled by a series of checkpoints [27], [28]. Activation of the spindle checkpoint delays the cell prior to anaphase onset to ensure the attachment of chromosomes via kinetochores to spindle microtubules [29]–[31]. Misorientation of the mitotic spindle elicited a delay in anaphase onset until the spindle was repositioned to the geometric center of the cell [11]. However, the perturbation of actin induced tilting of the mitotic spindle, which did not correlate with changes in the time required to reach anaphase onset [24]. Thus, it is currently unknown whether the orientation of the mitotic spindle effects spindle function and whether activation of the spindle checkpoint is involved.

The majority of these studies were conducted on two-dimensional (2D) substrates. However, most cells *in vivo* experience a three-dimensional (3D) arrangement of adhesive contacts, through the interaction with other cells and the surrounding extracellular matrix. The impact of a 3D microenvironment, in comparison to 2D microenvironments traditionally exploited in *in vitro* research, is a rapidly expanding area of research. This research exploits a number of cell culture platforms spanning from large multi-cellular aggregates in 3D matrices to the 3D presentation of cell adhesions at the single cell level [32]–[38]. At the single cell level the transition from a 2D planar presentation of cell adhesions to a 3D arrangement has been demonstrated to impact on the formation and composition of cell-matrix adhesions, assembly of the cytoskeleton, mechanosensing and metabolism [32]–[34]. Culturing cells within a 3D microenvironment composed of a basement membrane matrix caused increased tilting of the mitotic spindle [24], indicating that the 3D presentation of adhesive ligands also contributes to spindle orientation. Until recently there has been a lack of tools that enabled the investigation of cells cultured within structurally and biochemically controlled 3D microenvironments. In this work we used a microwell array, where the internal surfaces of the wells, but not the intervening plateau regions, are coated with fibronectin [39], [40]. Thus, this cell culture platform enables the 3D presentation of adhesive ligands in a controlled microenvironment in which shape and protein coating can be independently controlled [34]. Cell geometry affected spindle orientation and mitotic timings in the 3D environment of the microwells, highlighting the critical role of cell shape in guiding mitosis, regardless of the dimensionality of the microenvironment. The role of the actin cytoskeleton was altered by the 3D presentation of adhesive ligands, emphasizing the differences in cell behavior in 2D versus 3D cell culture platforms.

## Results

### Culturing cells in microwells enables cell shape to be controlled in 3D

**Figure 1:**
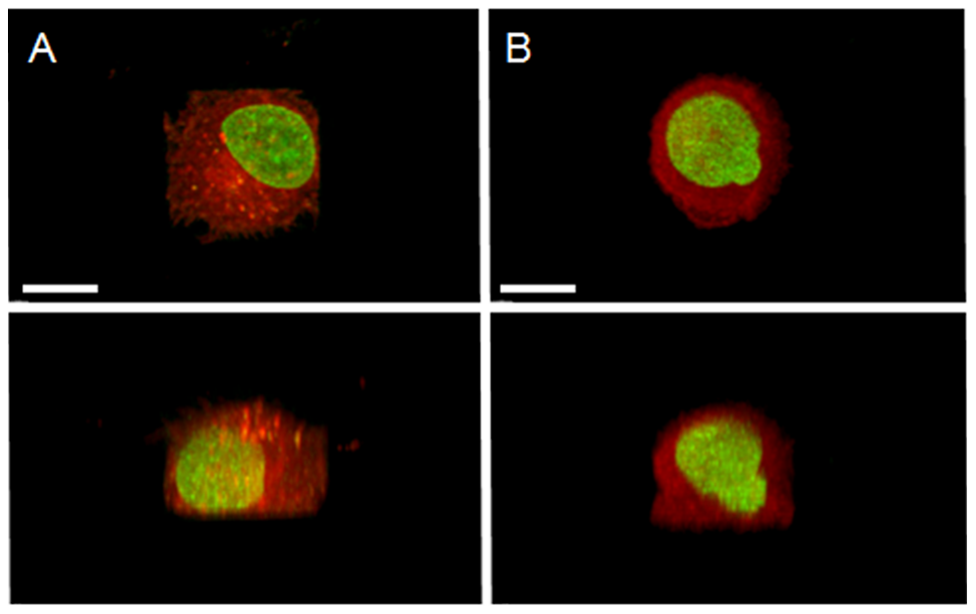
Control of 3D cell shape using a microwell cell culture platform. (A–B) HeLa cells (RFP-tubulin/GFP-H2B) were synchronized and cultured in square (A) or circular (B) microwells for 18 hours and imaged for nucleus (green) and cell outline (Vybrant DiD cell labeling solution, red). Images show the central xy slice of the cell (top) and the corresponding xz slice of the same cell (bottom); bars: 10 µm.

To control the cell geometry in a 3D environment, single HeLa cells were cultured in circular or square microwells coated with fibronectin, and compared to single cells cultured on homogenously coated 2D substrates. The surface area of the bottom of the microwells was 400 µm^2^ (20.0±0.4 µm in width for squares and 22.5±0.4 µm in diameter for circles) versus 11.0±1.5 µm deep, which was in the size range of single cell to ensure the shape of the cell was controlled by the shape of the microwell. As can be seen from the representative images in Fig. 1, single cells labeled with a membrane probe (Vybrant DiD cell labeling solution) adhered and filled the microwells to form square and circular cells (19.5±0.3 µm in diameter and 12.2±0.9 µm in height versus 22.1±0.8 µm in diameter and 12.3±0.6 µm in height, respectively). The circularity of the cells varied from 0.6±0.03 for cells cultured in squares microwells to 0.8±0.02 for cells cultured in circular microwells (p<0.001), indicating that cell geometry can be manipulated simply by adjusting the shape of the microwell.

### Cell shape and dimensionality of the cells' environment affects the orientation of the mitotic spindle

**Figure 2:**
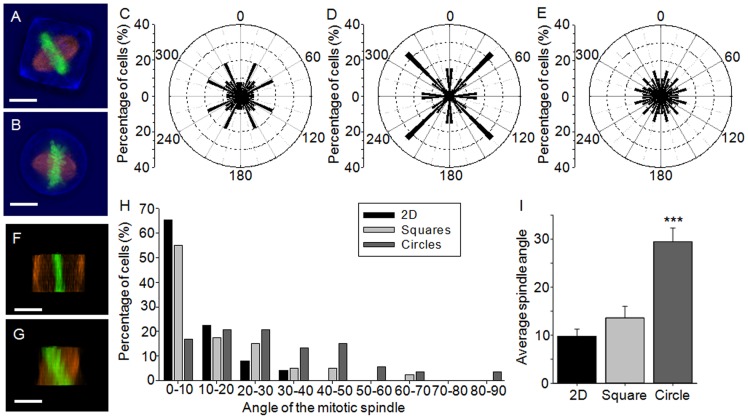
Effect of cell shape on the orientation of the mitotic spindle. The angle of mitotic spindle at metaphase was assessed in HeLa (GFP-H2B/RFP-tubulin or GFP-centrin-2/RFP-tubulin) cells cultured on the different platforms. To determine the orientation of the spindle parallel to the substrate plane, cells were imaged for microwell and cell shape (transmission channel, blue), DNA (green) and tubulin (red) after culture in (A) square microwells or (B) circular microwells; bars: 10 µm. Cells cultured (C) on 2D substrates or (E) within circular microwells possessed a random orientation of the mitotic spindle. (D) Conversely, a high proportion of the cells cultured in square microwells aligned the spindle along the long axis of the cell. To determine the orientation of the spindle perpendicular to the substrate plane, z stacks were reconstructed to obtain the xz view of the cell. Representative images are shown of cells with a (F) parallel versus (G) tilted orientation of the mitotic spindle; bars: 10 µm. (H) The angle of the spindle was dependent on whether cells were cultured on 2D homogenously coated substrates (black bars), or within square (light grey bars) or circular (dark grey bars) microwells. A higher percentage of cells cultured in circular microwells exhibited tilting of the mitotic spindle, in comparison to cells cultured in square microwells and on 2D substrates. (I) Furthermore, on average greater tilting was observed after cells were cultured in the circular microwells, while the mitotic spindle was aligned nearly parallel to the substrate in cells cultured in 2D or square microwells. Values represent spindle angle in degrees ± SEM. Key: *** p<0.001.

This cell culture platform was used to explore the effect of the 3D presentation of adhesive ligands and cell shape on spindle orientation. The analysis was conducted using HeLa cells expressing either RFP-tubulin/GFP-Histone-2B (Fig. 2, A and B) or RFP-tubulin/GFP-centrin-2 (Fig. S1), however no differences were observed between HeLa cells expressing either RFP-tubulin/GFP-H2B or RFP-tubulin/GFP-centrin-2 and therefore the data was grouped. The orientation of the mitotic spindle at metaphase was assessed parallel (xy plane) and perpendicular (xz plane) to the substrate plane. On 2D substrates the orientation of the long axis of individual HeLa cells varied greatly, which correlated with a random distribution of the mitotic spindle parallel to the substrate plane (Fig. 2C). Similarly, HeLa cells cultured in circular microwells did not exhibit a preferential orientation of the mitotic spindle (Fig. 2, B and E). Single cells cultured in square microwells preferentially orientated the mitotic spindle aligned along the longest axis of the cell, namely the diagonal of the square (33.3% aligned along the diagonal) (Fig. 2, A and D). Thus, similar to previous research [11], [13], the cell preferentially aligned the mitotic spindle along the long axis of the cell, regardless of the dimensionality of the microenvironment.

The perpendicular orientation of the mitotic spindle relative to the substrate plane (xz plane) at metaphase was also assessed (Fig. 2, F–I). In cells cultured on 2D substrates the average spindle angle was 9.8±1.5°, which correlates well with previous research where culturing cells on 2D substrates coated with fibronectin resulted in a parallel alignment of the mitotic spindle [24]. The average spindle angle observed in HeLa cells cultured in square microwells was 13.6±2.4°, which was not significantly different to the average angle observed in cells cultured on 2D substrates. The culture of single cells in circular microwells resulted in a random orientation of the mitotic spindle with an average spindle angle of 29.5±2.9° (p<0.001 relative to cells cultured on either 2D substrates or within square microwells). Thus, cell shape affected the perpendicular orientation of the mitotic spindle in the 3D environment of the microwells.

### Cell shape and dimensionality of the cells' environment affects progression through mitosis

Analysis of the orientation of the mitotic spindle at metaphase revealed that dimensionality and cell shape affected orientation; however the correlation between changes in the orientation of the mitotic spindle and their concurrent effect on its integrity and function is unclear. To explore the effect of cell geometry on the progression of the cell through mitosis, HeLa cells (GFP-H2B/RFP-tubulin) were synchronized and cultured within square or circular microwells or on 2D substrates and analyzed using time lapse fluorescent microscopy. Within the microwells, the cells rounded up (circularity  = 0.8±0.02 for cells cultured in both circular and square microwells and on 2D substrates), divided and successfully completed mitosis, indicating that their general physiology was not grossly perturbed (Movies S1 to 3). To assess the cells' progression through mitosis a number of key stages were identified, namely nuclear envelope breakdown (NEBD; identified by the condensation of chromatin into chromosomes and set at T = 0), late prometaphase (identified as the stage when the mitotic spindle had formed and the majority of the chromosomes had aligned at the spindle equator), metaphase (when complete alignment had occurred), anaphase (onset of chromosome-to-pole movement and start of spindle elongation) and cytokinesis (identified by the presence of the mid-body) (Fig. S2).

**Figure 3:**
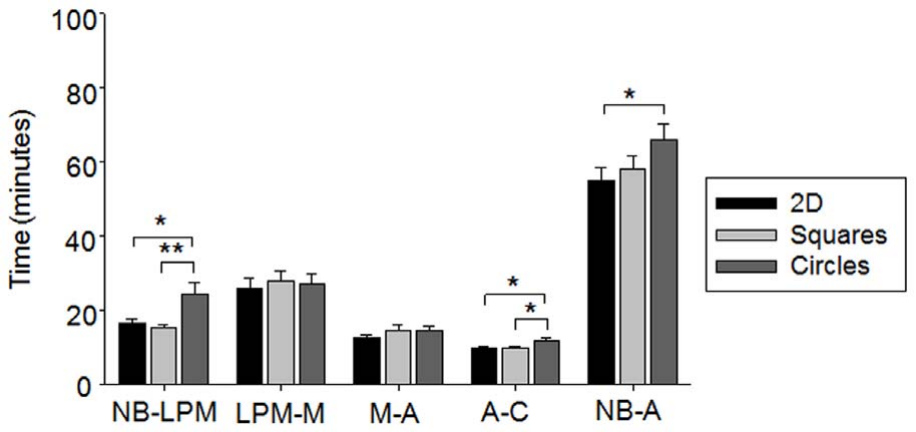
The effect of cell shape on the cells' progression through mitosis. HeLa cells (RFP-tubulin/GFP-H2B) were synchronized and cultured on 2D homogenously coated substrates (black bars) or within square (light grey bars) or circular (dark grey bars) microwells and assessed for the time required to progress through each stage in mitosis. Cells cultured in circular microwells took longer to progress through mitosis and specifically to reach late prometaphase, than cells cultured in square microwells or on 2D substrates. All values represent time in minutes ± SEM. Key: * p<0.05, ** p<0.01; NB  =  nuclear envelope breakdown, LPM  =  late prometaphase, M =  metaphase, A =  anaphase, C =  cytokinesis.

Single cells cultured within square microwells reached late prometaphase 15.4±0.8 min after NEBD (Fig. 3; Table 1). Progression from late prometaphase to metaphase lasted 27.9±2.7 min, while metaphase to anaphase and anaphase to cytokinesis lasted 14.6±1.7 and 9.9±0.5 min, respectively. Overall mitosis, from NEBD to anaphase, lasted 57.9±3.5 min. The mitotic timings were not significantly different to the timings observed for single cells cultured on 2D substrates homogenously coated with fibronectin. In contrast, significant differences were observed when single cells were cultured in circular microwells. An increased time between NEBD and late prometaphase (9.0±3.0 min increase, p = 0.006 and 7.7±3.1 min, p = 0.018, in comparison to square and 2D substrates, respectively) and anaphase and cytokinesis (1.7±0.9 min, p = 0.039 and 1.9±0.9 min, p = 0.039, in comparison to square and 2D substrates, respectively) was observed, and further the total time, between NEBD and anaphase, was also significantly increased (11.0±5.4 min, p = 0.046, in comparison to 2D substrates). Thus, altering the 3D cell morphology affected the progression of the cell through mitosis.

**Table 1:** Analysis of mitotic timings in single cells cultured on different cell culture platforms.

		Time (minutes)			
Cell culture platform	Treatment	NEBD to Spindle	NEBD to LPM	LPM to A	NEBD to A
**2D**		15.0±1.7	16.7±1.3	38.3±2.9	54.9±3.4
**2D Squares**		8.2±0.5	14.1±0.7	44.0±4.8	58.1±5.0
**3D Squares**		14.0±0.9	15.4±0.8	42.4±4.4	57.9±3.5
**3D Circles**		13.3±0.9	24.4±3.0	41.5±4.0	65.9±4.2
**3D Squares**	scrambled siRNA	9.9±0.9	17.1±1.2	40.9±5.6	57.6±5.7
**3D Circles**	scrambled siRNA	11.0±1.0	25.5±1.9	52.0±7.3	77.5±7.8
**3D Squares**	Mad2 siRNA	10.2±1.1	14.2±1.1	12.0±2.1	26.2±2.2
**3D Circles**	Mad2 siRNA	8.6±0.7	12.8±0.6	10.5±1.4	23.2±1.4
**2D Squares**	Lat A	8.1±0.5	13.8±0.8	22.5±3.5	36.3±3.8
**3D Squares**	Lat A	14.6±1.1	25.3±1.8	46.1±5.0	71.4±5.4
**3D Circles**	Lat A	16±1.6	24.0±1.5	44.0±4.4	68.0±4.8

### Differential activation of the spindle checkpoint is responsible for the shape dependent effects on mitotic progression

**Figure 4:**
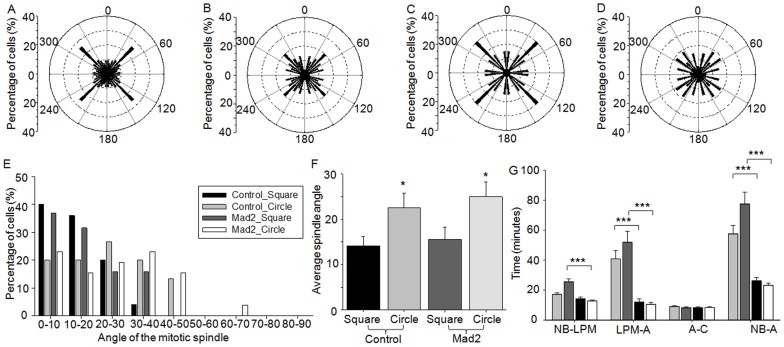
The role of spindle checkpoint activation in the shape dependent differences in mitosis. HeLa (GFP-H2B/RFP-tubulin) cells were assessed for the parallel orientation of the mitotic spindle at metaphase after transfection with (A and B) scrambled siRNA or (C and D) Mad2 siRNA and culture in (A and C) square or (B and D) circular microwells to determine the role of spindle checkpoint activation. Cells cultured in circular microwells showed a random orientation of the mitotic spindle, whilst cells cultured in square microwells predominately aligned the spindle along the long axis of the cell, regardless of the depletion of Mad2. Similarly, the depletion of Mad2 had little effect on (E) the distribution and (F) average spindle orientation perpendicular to the substrate plane. Values represent spindle angle in degrees ± SEM. (G) Cells transfected with siRNA for Mad2 and cultured in circular microwells (white bars) took significantly less time to reach late prometaphase than cells transfected with control siRNA (dark grey bars), indicating that the shape dependent effect on the time required to reach late prometaphase was due to differences in spindle checkpoint activation. All values represent time in minutes ± SEM. Key: * p<0.05, *** p<0.001; NB  =  nuclear envelope breakdown, LPM  =  late prometaphase, M =  metaphase, A =  anaphase, C =  cytokinesis.

To test whether the differences in spindle orientation and mitotic timings were due to the differential activation of the spindle checkpoint, HeLa cells (GFP-H2B/RFP-tubulin) were transfected with small interfering RNA (siRNA) against the spindle checkpoint protein, Mad2, to abolish mitotic arrest [41]. To discount any possible detrimental effects of the transfection procedure itself, the Mad2-depleted cells were compared to cells transfected with scrambled siRNA, which exhibited no significant differences in spindle orientation and mitotic timings, in comparison to unperturbed cells. Cells cultured in square microwells orientated the mitotic spindle along the diagonal of the microwell (25.9 to 30.0%) and cells cultured circular microwells exhibited a random orientation in the presence of either scrambled siRNA or siRNA directed against Mad2 (Fig. 4, A to D). The average orientation of the mitotic spindle perpendicular to the substrate plane (xz plane) was also not significantly affected by the depletion of Mad2 (14.2±2.0° versus 15.6±2.7° for scrambled siRNA transfected or Mad2-depleted cells cultured in square microwells and 22.5±3.3° versus 24.9±3.3° for cells cultured in circular microwells) (Fig. 4, E and F). Therefore, the disruption of spindle checkpoint activation did not correlate with the shape dependent differences in spindle orientation.

As expected, progression through mitosis was greatly accelerated in Mad2-depleted cells (Fig. 4G; Table 1) [41], [42], after culture in both square and circular microwells. In cells cultured in circular microwells the progression from NEBD to late prometaphase was also accelerated, (12.7±2.0 min, p<0.001, in comparison to scrambled siRNA transfected cells), which was not significantly different to the time required after culture in square microwells. Consequently, the differences in the onset of late prometaphase observed between cells cultured in square versus circular microwells was abolished by the depletion of Mad2, indicating that this shape dependent effect was due to differences in the activation of the spindle checkpoint.

### Centrosome separation and spindle formation were not affected by cell shape or dimensionality

Prior to the metaphase to anaphase transition the cell must complete two processes; the formation of a bipolar spindle and the alignment of the chromosomes at the metaphase plate. The formation of the bipolar spindle requires the separation of the centrosomes to form two separate microtubule organizing centers (MTOCs) [43]. This separation of the centrosomes can occur via two distinct pathways [44]–[46]; either an orthogonal alignment of the centrosomes is achieved prior to NEBD, termed the prophase pathway, or after NEBD, termed the prometaphase pathway. To determine whether changes in the time required for centrosome separation and spindle formation were responsible for the shape dependent differences in mitotic timings the position of the centrosomes at NEBD versus the time required for spindle formation was examined on the different cell culture platforms (Fig. S3). In 33 to 34% of cells cultured on 2D substrates or within square microwells the centrosomes were positioned on the opposite sides of the nucleus at the initiation of NEBD, in comparison to 47.4% of cells cultured in circular microwells. However, the time required to form the spindle was not significantly different for the different cell culture platforms assessed, indicating that the shape dependent effects observed were not due to differences in centrosome separation and spindle formation. Consequently, we propose that the differences in mitotic timings observed in circular versus square microwells can be attributed to differences in the time required to align the chromosomes at the metaphase plate.

### Actin cytoskeleton varies on the different cell culture platforms

**Figure 5:**
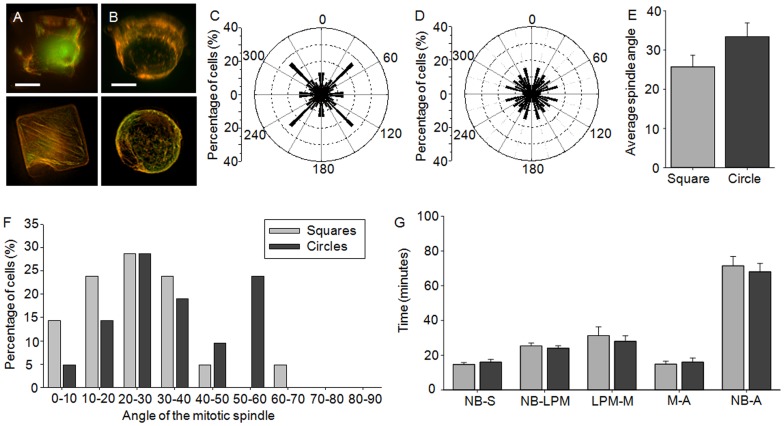
The role of the actin cytoskeleton in the shape dependent differences in mitosis. HeLa (YFP-paxillin) cells were cultured for 18 hours in either (A) square microwells or (B) circular microwells and imaged during interphase for actin (red) and paxillin (green); bars: 10 µm. HeLa (GFP-H2B/RFP-tubulin) cells were assessed for the parallel orientation of the mitotic spindle at metaphase after culture in (C) square or (D) circular microwells and treatment with latrunculin A for 1 hour prior to mitosis. Cells cultured in circular microwells showed a random orientation of the mitotic spindle, whilst cells cultured in square microwells predominately aligned the spindle along the long axis of the cell. (E) The inhibition of actin polymerization in HeLa (GFP-H2B/RFP-tubulin) cells lead to increased tilting in cells cultured in the square microwells. Values represent spindle angle in degrees ± SEM. (F) Perturbation of actin polymerization in cells cultured in square microwells also increased the distribution of spindle orientation, but had little effect on cells cultured in circular microwells. (G) The perturbation of the actin cytoskeleton negated the differences in mitotic timings observed between cells cultured in square microwells (light grey bars) and cells cultured in circular microwells (dark grey bars). All values represent time in minutes ± SEM. NB  =  nuclear envelope breakdown, S =  spindle formation, LPM  =  late prometaphase, M =  metaphase, A =  anaphase.

Actin, and its associated motor proteins, contribute to cell rounding prior to mitosis [47], centrosome separation [46], [48]–[50] and the formation of the cleavage furrow leading to cytokinesis [50]–[52]. The directionality of intracellular tractional forces during interphase, imposed either via actomyosin contractility or externally applied forces, also guides the parallel and perpendicular orientation of the mitotic spindle [13], [20], [24], [53]. Thus, we wished to explore whether changes in the actin cytoskeleton were responsible for the shape dependent differences observed. To assess this, HeLa cells expressing YFP-paxillin were cultured within the microwells and retrospectively labeled with TRITC phalloidin to visualize the actin cytoskeleton in interphase cells. When HeLa cells were cultured within circular microwells the actin cytoskeleton and associated plaque protein, paxillin, were predominately diffuse throughout the ventral cell surface (Fig. 5B). However, at the lateral cell surfaces small fibrillar adhesions associated with paxillin, were observed running perpendicular to the substrate plane. Short lateral stress fibers were also observed in cells cultured in square microwells (Fig. 5A). In addition, fibrillar adhesions were observed at the ventral cell surface, which typically spanned the diagonal axis of the cell and were associated with paxillin at the cell periphery, indicating the formation of focal adhesions [54]. Thus, cell morphology changes correlated with changes in the actin cytoskeleton, similar to cells cultured on 2D patterns [14], [16].

### Actin cytoskeleton is responsible for the shape dependent effect on spindle orientation and mitotic timings

To determine the contribution of these changes in the actin cytoskeleton on the shape dependent effect observed in the microwells, actin polymerization was disrupted by the addition of latrunculin A [13], [50], [53]. Cell rounding prior to mitosis was unaffected by the disruption of the actin cytoskeleton (circularity  = 0.9±0.01 versus 0.8±0.01 in the absence of latrunculin A, for cells cultured in both circular and square microwells). The orientation of the mitotic spindle parallel to the substrate plane was unaffected by the perturbation of the actin cytoskeleton. Cells within square microwells preferentially orientated their spindle along the diagonal (26.3% aligned along the diagonal) (Fig. 5C), while cells cultured within circular microwells still exhibited a random distribution (Fig. 5D). The addition of latrunculin A slightly increased tilting but had little effect on the average orientation of the mitotic spindle perpendicular to the substrate plane in cells cultured in circular microwells (Fig. 5, E and F). Conversely, in cells cultured in square microwells it resulted in an increase in the average angle of the mitotic spindle from 13.6±2.4° in untreated cells to 25.7±3.0° upon the addition of latrunculin A (p = 0.002) (compare Fig. 5, E and F to Fig. 2, H and I). In the presence of latrunculin A, cells initiated NEBD and proceeded through the preliminary stages of mitosis as normal, however many failed to complete cytokinesis, as expected given the acknowledged importance of actin for the formation of the contractile ring [50], [51]. Consequently, this stage was omitted from the analysis of mitotic timings. Cells cultured in circular microwells were unaffected by the inclusion of latrunculin A; no significant differences were observed in comparison to untreated cells at any stage during mitosis (compare Fig. 5G to Fig. 3; Table 1). In cells cultured in square microwells the progression from NEBD to late prometaphase and from NEBD to anaphase was significantly increased, in comparison to untreated cells (9.9±1.9 min, p<0.001 and 13.5±6.4 min, p = 0.04, respectively). Consequently, no significant differences were observed in the presence of latrunculin A in cells cultured in square versus circular microwells. Hence, the inhibition of actin polymerization abolished the shape dependent differences in mitotic timings.

### Effect of actin on mitotic timings and orientation of the mitotic spindle in cells cultured on 2D square patterns

**Figure 6:**
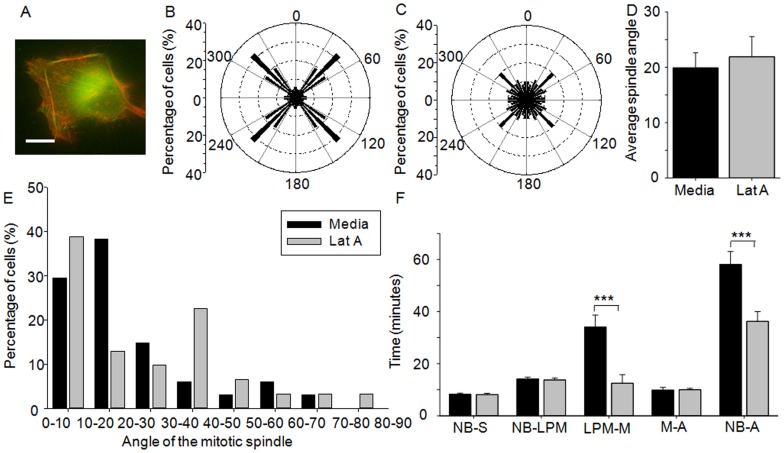
The effect of the actin cytoskeleton on mitosis after culture on 2D square patterned substrates. (A) HeLa cells (YFP-paxillin) were cultured on 2D square patterns for 18 hours and imaged during interphase for actin (red) and paxillin (green). HeLa cells (RFP-tubulin/GFP-H2B) were assessed for the angle of the mitotic spindle in the xy plane at metaphase after culture on 2D square patterns and treatment with (B) media or (C) latrunculin A. A high proportion of the cells cultured on square patterns aligned the mitotic spindle along the diagonal of the cell, which was reduced by the addition of latrunculin A. (D) Conversely the addition of latrunculin A had little effect on the average orientation of the mitotic spindle perpendicular to the substrate plane. Values represent spindle angle in degrees ± SEM. (E) Furthermore, the addition of latrunculin A had little effect on the distribution of the spindle orientation. (F) Cells treated with latrunculin A (light grey bars) took significantly less time to progress from late prometaphase to metaphase and to complete mitosis than untreated cells (black bars). All values represent time in minutes ± SEM. Key: *** p<0.001; NB  =  nuclear envelope breakdown, S =  spindle formation, LPM  =  late prometaphase, M =  metaphase, A =  anaphase.

To elucidate the differential contribution of cell shape and the dimensionality of the microenvironment on spindle orientation and mitotic timings, single cells were cultured on 2D square patterns (30 μm ×30 μm) (Movie S4 and Fig. 6A). Similar to cells cultured within square microwells, there was a significant increase in the circularity of the cell prior to mitosis (circularity was 0.5±0.03 versus 0.9±0.002, p<0.001, in interphase versus mitotic cells, respectively). Cells preferentially orientated the mitotic spindle along the diagonal of the square (32.4% aligned along the diagonal of the square) (compare Fig. 6B to Fig. 2D). Similar to cells cultured in square microwells, the inclusion of latrunculin A did not affect cell rounding prior to mitosis (circularity  = 0.9±0.01). However, as expected, treatment of the cells with latrunculin A reduced the proportion of the cells with the mitotic spindle aligned along the diagonal of the square (Fig. 6C) [13]. This differed from the 3D case, where the disruption of the actin cytoskeleton had little effect on the orientation of the mitotic spindle parallel to the substrate plane (xy plane) (Fig. 5C). The average orientation of the mitotic spindle perpendicular to the substrate plane (xz plane) was not significantly different to the orientation observed in cells cultured in square microwells (compare Fig. 6, D and E to Fig. 2, H and I). The addition of latrunculin A slightly increased the extent of tilting observed, but did not affect the average spindle orientation. Thus, in contrast to cells cultured in square microwells, the disruption of the actin cytoskeleton had a greater impact of the orientation in the xy plane, than the orientation in the xz plane.

The culture of single cells on 2D square patterns, as opposed to within 3D microwells, had no effect on the progression from NEBD to late prometaphase or the total time required for the cell to progress from NEBD to anaphase (Compare Fig. 5G and 6F; Table 1). Conversely, the time required for spindle formation and progression from metaphase to anaphase was significantly decreased (4.7±0.9 min decrease, p<0.001 and 4.7 min decrease, p = 0.017, respectively), indicating that changes in dimensionality alone can alter progression through mitosis. Centrosome separation occurred via the prophase pathway in a higher proportion of cells cultured on 2D patterns, in comparison to cells cultured in 3D microwells (62.9% versus 34.0%), indicating that faster spindle formation correlated, in this instance, with faster centrosome separation. Disruption of the actin cytoskeleton after culture on 2D square patterns greatly reduced the time between late prometaphase and metaphase (21.6±5.6 min decrease, p<0.001), which resulted in a decreased time between NEBD and anaphase (21.9±6.3 min decrease, p<0.001). This accelerated progression was consistently observed and indicates that the inhibition of actin helped mature the metaphase plate. This result is somewhat surprising, however in *Xenopus laevis* XL2 cultured cells the addition of latrunculin A substantially decreased metaphase, although in that instance it correlated with an increase in prometaphase [50]. This is in contrast to the 3D case, where the inhibition of actin slowed the progression of the cell through mitosis.

## Discussion

The formation and positioning of the mitotic spindle is critical in determining the placement of cytokinesis, and consequently defines the position and fate of the resultant daughter cells. Previous research has provided intriguing insights into the role of cell shape and adhesive environment in establishing the orientation of the mitotic spindle [11], [13], [24]. However, few researchers have attempted to extend this knowledge into a 3D microenvironment, in part due to the lack of appropriate cell culture platforms. In this study, we exploited a microwell platform that allows cells to be cultured in a constrained 3D environment, enabling the cell shape to be defined by the shape of the microwell. Consequently, it was possible to examine the effects of cell shape and the 3D presentation of adhesive ligands on the formation and alignment of the mitotic spindle during mitosis.

### 3D cell shape affects spindle orientation and mitotic timings

Cell shape affected mitosis in the reductionist 3D environment of the microwell, indicating that cell shape is a critical parameter in both 2D and 3D [10], [11], [13]. In cells cultured on 2D cell culture platforms the spatial distribution of the adhesive environment, and consequently actin cytoskeleton, during interphase predetermines the orientation of the mitotic spindle via the formation of retraction fibers [13], [20]. Similarly, cells cultured in square microwells exhibited stress fibers aligned along the diagonal of the cell during interphase, which correlated with the orientation of the spindle. In cells cultured in circular microwells diffuse actin staining correlated with greater tilting of the mitotic spindle and increased the time required for the alignment of chromosomes at the metaphase plate. Thus, the absence of alignment imparted by the adhesive environment during interphase results in increased tilting of the mitotic spindle and prolonged activation of the spindle checkpoint.

The correlation between the orientation of the mitotic spindle and the impact on its function and integrity of the mitotic spindle has rarely been explored. Using this cell culture platform it was revealed that the orientation of the mitotic spindle did indeed correlate with changes in mitotic timings. The molecular mechanisms linking the mitotic spindle orientation and function is currently unclear, however previous research indicates that misorientation of the spindle can occur in the absence of an effect on spindle checkpoint activation [24]. Furthermore, the disruption of spindle checkpoint activation, through the depletion of Mad2, affected mitotic timings in a shape dependent manner, but had no impact on spindle orientation. We therefore postulate that a lack of intracellular directionality causes a misorientation of the spindle and that it leads to activation of the spindle checkpoint, resulting in a delay in mitotic timing. Our data shows that spindle orientation is not caused by the activation of the spindle checkpoint; however whether the converse is true is not clear at the moment. We observe a correlation between an extended checkpoint activation and spindle orientation, however future experiments will be necessary to determine whether there is a direct causal relationship.

### The effect of dimensionality on spindle orientation and mitotic timings

The 3D organization of adhesive contacts has been demonstrated to impact on many cell responses, including adhesion, morphology, proliferation and metabolism [24], [32]–[34]. Typically, spindle orientation is explored in cells cultured on 2D substrates, either homogenously coated or patterned with adhesive proteins [13], [20], [24], which are unsuitable for the exploration of the effect of dimensionality. Conversely, the microwell cell culture platform presented in this study is ideal for the study of the effect of dimensionality in a controlled microenvironment. In particular, the comparison of cells cultured in 3D square microwells versus cells cultured on 2D square patterns allowed the elucidation of the effect of dimensionality, independently of changes in cell shape.

The actin cytoskeleton formed in cells cultured on 2D squares patterns and in square microwells was qualitatively very similar, and the orientation of the mitotic spindle during mitosis correlated with the directionality of the actin cytoskeleton observed during interphase in both cell culture platforms. This indicates that in the 3D microenvironment of the microwell, similar to the 2D scenario, the spatial distribution of intracellular traction forces and adhesive environment is critical in determining spindle orientation. However, a differential dependency on the organization of actin was observed depending on the dimensionality of the cells microenvironment. As expected, on 2D square patterns the perturbation of actin impacted on the orientation of the mitotic spindle parallel to the substrate plane [13], but in contrast to previous research [24], it did not affect orientation perpendicular to the substrate plane and accelerated the progression of the cell through mitosis. Cells cultured in 3D microwells exhibited the same alignment of the mitotic spindle as cells cultured on 2D square patterns, however the perturbation of actin disrupted the orientation of the mitotic spindle perpendicular, but not parallel to the substrate plane, and resulted in a delay in mitotic timings. Hence, it can be concluded that the role of actin in orientation of the mitotic spindle varies depending on the dimensionality of the cells' microenvironment. The presence of short lateral stress fibers in cells cultured in 3D microwells, but not on 2D patterns indicates that spatial distribution of interphase adhesions differs between the two cell culture platforms. This relocation of cellular adhesions along the z axis may be responsible for the differences observed. This supports the rapidly expanding evidence that 2D cell culture platforms may not accurately predict cell behavior in their native 3D microenvironment, thus highlighting the necessity of 3D cell culture platforms. One critical challenge for the future will be the use of controlled 3D cell culture platforms to determine the molecular mechanisms responsible for these intriguing differences in cell behavior.

## Materials and Methods

### Fibronectin isolation

Human plasma fibronectin (Fn) was isolated from fresh human plasma (Swiss Red Cross) using gelatin-sepharose chromatography and established methods [55], as described in [40].

### Substrate fabrication

Arrays of microwells with different geometries (squares, width 20 µm and circles, diameter 22.5 µm) and 10 µm deep were fabricated in PDMS, as previously described [40]. Briefly, microstructures were created in silicon using standard photolithography with the negative photoresist SU8 (MicroChem, Germany) and replicated into a polydimethylsiloxane master (PDMS, Sylgard 184, Dow Corning, Switzerland 1∶10 w/w curing agent to pre-polymer). After fluorosilanization of the PDMS master, it could be repeatedly used to create thin film PDMS replicates on thin glass cover slips (Menzel-Gläser, Germany, strength 0, approx. 100 µm thickness). 2D samples were prepared by molding thin PDMS films onto thin glass cover slips. Samples were glued into the bottom of a Petri dish into which a hole was previously drilled to facilitate cell culture and imaging of the samples.

### Substrate functionalization

The 2D substrates and PDMS microwells were rendered hydrophilic by exposure to air plasma at 0.8 mbar for 35 s (PDC-002, Harrick Scientific, USA). Thereafter the homogenously 2D substrates were coated with fibronectin (25 μgml^−1^, 1 hour). After air plasma treatment the plateau areas of the 3D microwell arrays were passivated with poly(L-lysine)-*graft*-poly(ethylene glycol) (PLL(20 kDa)-*g*-[3.4]-PEG(2 kDa); (PLL-*g*-PEG), SuSoS, Switzerland) using an inverted microcontact printing technique [40]. Briefly, a flat PLL-*g*-PEG loaded hydrogel was placed on the structured substrate resulting in conformal contact between the stamp and the plateau, but not the insides of the well. The contact transferred PLL-*g*-PEG to the plasma treated PDMS plateau surface, rendering it resistant to protein and vesicle adsorption [56], [57]. Subsequently, the internal surfaces of the individual microwells were functionalized with fibronectin (25 μgml^−1^, 1 hour) and washed with PBS. To generate the 2D fibronectin patterns (30 μm×30 μm squares) a PDMS stamp was inked with a 25 μgml^−1^ fibronectin solution, 50% of which was labeled with Alexa 488 (Molecular Probes, Switzerland), for 20 min, dried, and placed in contact with an untreated glass coverslip. After removal of the stamp, the printed coverslip was immersed in PBS containing 25 mgml^−1^ PLL-*g*-PEG for 1 h at room temperature. The coverslip was then washed in PBS before cell deposition.

### Cell culture

HeLa-B (human adenocarcinoma epithelial cell line) cells, stably expressing either GFP-Histone-2B and RFP-tubulin, GFP-centrin-2 and RFP-tubulin or YFP-paxillin [41], [58], were maintained in Dulbecco's modified Eagle's medium (DMEM, Gibco, Switzerland) supplemented with foetal calf serum (10% (v/v), Gibco), penicillin (100 Uml^−1^, Gibco), streptomycin (100 μgml^−1^, Gibco) and amphotericin (0.625 μgml^−1^, Gibco) and grown in a humidified atmosphere (95% (v/v) air, 5% (v/v) carbon dioxide at 37°C) to 90% confluence. HeLa-B (GFP-Histone-2B/RFP-tubulin) cells were cultured in the presence of puromycin (0.5 μgml^−1^, Sigma-Aldrich, Switzerland) and HeLa-B (GFP-centrin-2/RFP-tubulin) cells were cultured in the presence of puromycin (1 μgml^−1^) and gentamicin sulfate (500 μgml^−1^, Invitrogen, Switzerland).

### Synchronization

Cells (both HeLa-B (GFP-Histone-2B/RFP-tubulin) cells and HeLa-B (GFP-centrin-2/RFP-tubulin) cells) were synchronized at the G1/S boundary using a double thymidine block, specifically the first block was performed for 16 hours (2 mM thymidine, Sigma-Aldrich), followed by a 9 hour release (30 µM deoxycytidin, Sigma-Aldrich) and finally a second block of 16 hours (2 mM thymidine). Cells were released from the second block and passaged with trypsin/EDTA (0.02% (w/v)/0.05% (w/v), Gibco) and seeded onto substrates at 1×10^4^ cells per ml. Cells were allowed to adhere within the microwells and on 2D square patterns for 30 minutes, after which the unbound cells on the non-adhesive background were removed by gentle pipetting. Cells were subsequently rested for 10 hours before imaging.

### RNA interference and inhibitor treatment

HeLa-B (GFP-Histone-2B/RFP-tubulin) cells were transfected after the first block using Oligofectamine (Invitrogen) with 20 nM siRNA duplexes for Mad2 (targeted sequence: AAG AGT CGG GAC CAC AGT TTA) or the scrambled control (targeted sequence: AAG GAC CTG GAG GTC TGC TGT) for 24 hours in modified essential media (Sigma-Aldrich) supplemented with foetal calf serum (10% (v/v)), penicillin (100 Uml^−1^), streptomycin (100 μgml^−1^) and amphotericin (0.625 μgml^−1^) and puromycin (0.5 μgml^−1^). Latrunculin A (1 µM, Sigma-Aldrich) was added to HeLa-B (GFP-Histone-2B/RFP-tubulin) cells 9 hours after cell adhesion in microwells and 1 hour before entry into mitosis.

### Time lapse microscopy

For live cell imaging, cells were monitored in Leibovitz's L-15 medium supplemented with fetal calf serum (10% (v/v)), penicillin (100 Uml^−1^), streptomycin (100 μgml^−1^) and amphotericin (0.625 μgml^−1^). Time points, comprised of 10 z sections 1 µm apart, were acquired every 4 minutes for 8 hours with a 63× objective lens (1.4NA DIC oil PlanApo, Olympus) and a camera (CoolSNAP HQ; Roper Scientific) on an imaging system (DeltaVision Core, Applied Precision) fitted with a 37°C environmental chamber. Image stacks were deconvolved and quantified with SoftWorx (Applied Precision, LLC) and mounted in figures using Imaris and Photoshop. The orientation of the mitotic spindle parallel and perpendicular to the substrate plane was measured in metaphase cells and defined as the angle of the chromosomes relative to the side of the microwell or substrate plane, respectively. This is with the exception of cells treated with siRNA, which due to the lack of complete chromosome congression at metaphase, were analyzed at anaphase for spindle orientation.

### Retrospective fluorescent microscopy

HeLa-B (GFP-H2B/RFP-tubulin) cells were cultured in square or circular microwells for 18 hours before fixing by the addition of paraformaldehdye (4% (w/v) in PBS, Sigma-Aldrich, Switzerland) for 15 min and counter-stained by the addition of Vybrant DiD cell labeling solution (5 µM, 30 minutes, Molecular Probes). Images were subsequently captured on a Zeiss LSM510 confocal laser scanning microscope (Carl Zeiss AG, Germany) equipped with a 63× objective lens (1.4NA oil DIC Plan-Apochromat). Image stacks were captured using Zen software (Carl Zeiss AG) and mounted in figures using Imaris and Adobe Photoshop.

HeLa-B (YFP-paxillin) cells were used to visualize the actin cytoskeleton after culture in the different shaped microwells and 2D square patterns. Consequently, HeLa-B (YFP-paxillin) cells were cultured on the different cell culture platforms for 18 hours before fixing for 3 min in warm 3% paraformaldehdye with Triton X-100 (0.5% (v/v), Fluka-Chemie AG) followed by 3% paraformaldehdye alone for an additional 40 min. Finally, HeLa-B (YFP-paxillin) cells were stained with phalloidin-TRITC (1∶200 dilution, Molecular Probes) and imaged using a Zeiss Live Cell Station microscope (Carl Zeiss AG) equipped with a camera (Hamamatsu) and 63× objective lens (1.4NA Oil DICIII Plan Apochromat). Image stacks were captured using Metamorph (Molecular Devices, Sunnyvale, CA) and deconvolved with Huygens Remote Manager and mounted in figures using Imaris and Adobe Photoshop.

### Statistical analysis

At least 30 single cells were counted per condition across four independent experiments. Statistical analysis was calculated using Student's unpaired two-way t-tests.

## Supporting Information

Figure S1 **Assessment of the orientation of the mitotic spindle.** HeLa cells (GFP-centrin-2/RFP-tubulin) were synchronized and cultured on 2D substrates or within square microwells (A) or circular (B) microwells. Cells were imaged for centrin-2 (green), tubulin (red) and microwell outline (transmission, blue) and assessed at metaphase for the orientation of the mitotic spindle in the xy plane (A and B) and xz plane (C and D); bars: 10 µm.
(TIF)

Figure S2 **Assessment of the effect of cell shape on mitotic timings.** HeLa cells (RFP-tubulin/GFP-H2B) were synchronized and cultured on i) 2D substrates, ii) 3D square microwells or iii) 3D circular microwells for 10 hours before imaging using time lapse microscopy. Cells were imaged for DNA (green), tubulin (red) and microwell outline (transmission, blue) and assessed at different stages during mitosis, specifically (A) NEBD, (B) late prometaphase, (C) metaphase, (D) anaphase and (E) cytokinesis; bars: 10 µm.
(TIF)

Figure S3 **Cell shape did not impact on centrosome separation and spindle formation.** (A–B) HeLa cells (RFP-tubulin/GFP-H2B) were synchronized and cultured on the different cell culture platforms and assessed for the position of the centrosomes at NEBD and subsequent spindle formation. Cells were imaged for DNA (green), tubulin (red) and cell outline (transmission, blue); bars: 10 µm. Cells initiated the separation of their centrosomes either (A) during prophase, resulting in centrosomes orthogonal at NEBD or (B) during prometaphase, resulting in centrosomes at the same side of the nuclear envelope at NEBD. (C) Cells with the centrosomes positioned on opposite sides of the nuclear envelope at NEBD were quicker at forming the spindle, regardless of the substrate upon which the cells were cultured. Key: *** p<0.001.
(TIF)

Video S1 **Assessment of the effect of cell shape on mitosis in cells cultured on 2D substrates.** HeLa (GFP-H2B/RFP-tubulin) cells were synchronized and cultured on 2D substrates for 10 hours before imaging for DNA (green) and tubulin (red) using time lapse microscopy (Delta Vision imaging system). Time points, comprised of 10 z sections 1 µm apart, were acquired every 4 minutes.
(AVI)

Video S2 **Assessment of the effect of cell shape on mitosis in cells cultured in square microwells.** HeLa (GFP-H2B/RFP-tubulin) cells were synchronized and cultured in 3D square microwells for 10 hours before imaging for DNA (green), tubulin (red) and microwell outline (transmission, blue) using time lapse microscopy (Delta Vision imaging system). Time points, comprised of 10 z sections 1 µm apart, were acquired every 4 minutes.
(AVI)

Video S3 **Assessment of the effect of cell shape on mitosis in cells cultured in circular microwells.** HeLa (GFP-H2B/RFP-tubulin) cells were synchronized and cultured in 3D circular microwells for 10 hours before imaging for DNA (green), tubulin (red) and microwell outline (transmission, blue) using time lapse microscopy (Delta Vision imaging system). Time points, comprised of 10 z sections 1 µm apart, were acquired every 4 minutes.
(AVI)

Video S4 **Assessment of the effect of cell shape on mitosis in cells cultured on 2D patterns.** HeLa (GFP-H2B/RFP-tubulin) cells were synchronized and cultured on 2D square patterns for 10 hours before imaging for DNA (green) and tubulin (red) using time lapse microscopy (Delta Vision imaging system). Time points, comprised of 10 z sections 1 µm apart, were acquired every 4 minutes.
(AVI)
