# Supplementary figures and images for: Effect of Cell Shape and Dimensionality on Spindle Orientation and Mitotic Timing
Source: PLoS One. 2013 Jun 18;8(6):e66918. doi: 10.1371/journal.pone.0066918 (PMC3688943; doi:10.1371/journal.pone.0066918)

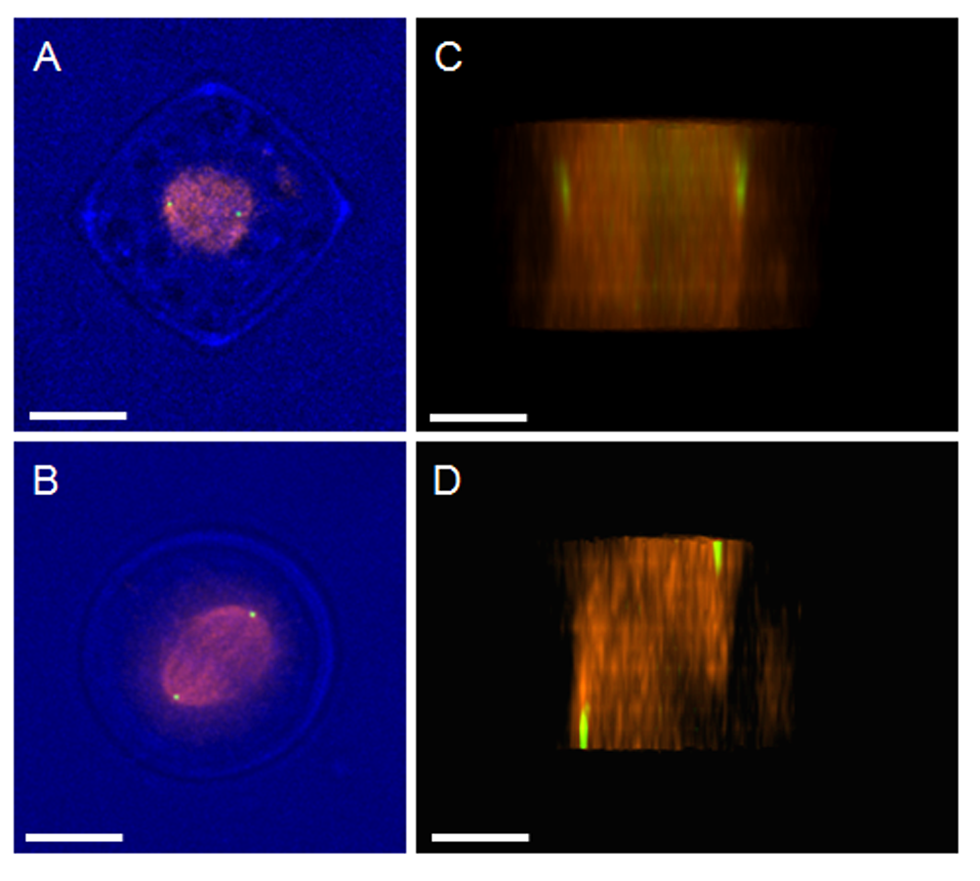

Supplement: Figure S1 — Assessment of the orientation of the mitotic spindle. HeLa cells (GFP-centrin-2/RFP-tubulin) were synchronized and cultured on 2D substrates or within square microwells (A) or circular (B) microwells. Cells were imaged for centrin-2 (green), tubulin (red) and microwell outline (transmission, blue) and assessed at metaphase for the orientation of the mitotic spindle in the xy plane (A and B) and xz plane (C and D); bars: 10 µm. (TIF) [file pone.0066918.s001.tif]

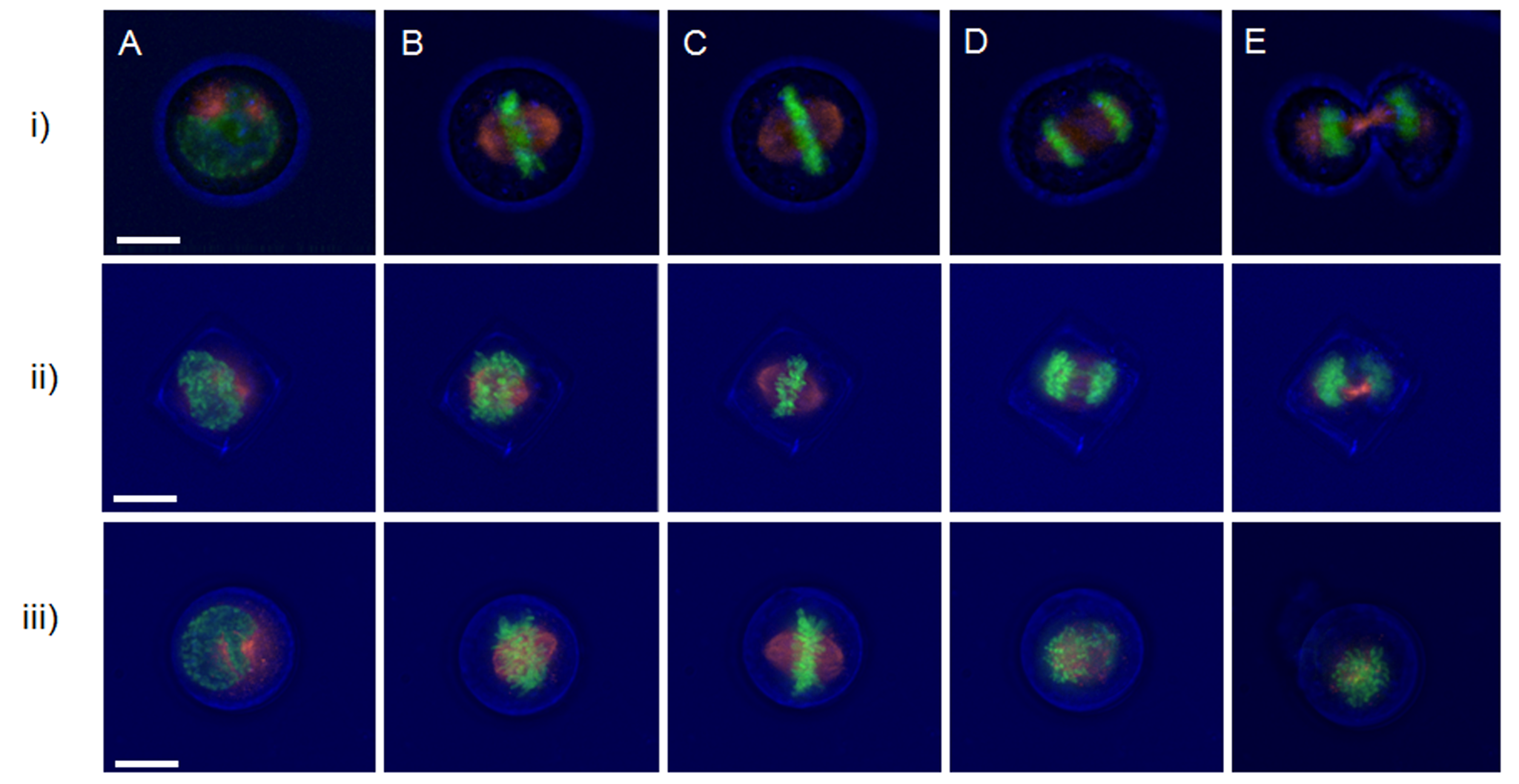

Supplement: Figure S2 — Assessment of the effect of cell shape on mitotic timings. HeLa cells (RFP-tubulin/GFP-H2B) were synchronized and cultured on i) 2D substrates, ii) 3D square microwells or iii) 3D circular microwells for 10 hours before imaging using time lapse microscopy. Cells were imaged for DNA (green), tubulin (red) and microwell outline (transmission, blue) and assessed at different stages during mitosis, specifically (A) NEBD, (B) late prometaphase, (C) metaphase, (D) anaphase and (E) cytokinesis; bars: 10 µm. (TIF) [file pone.0066918.s002.tif]

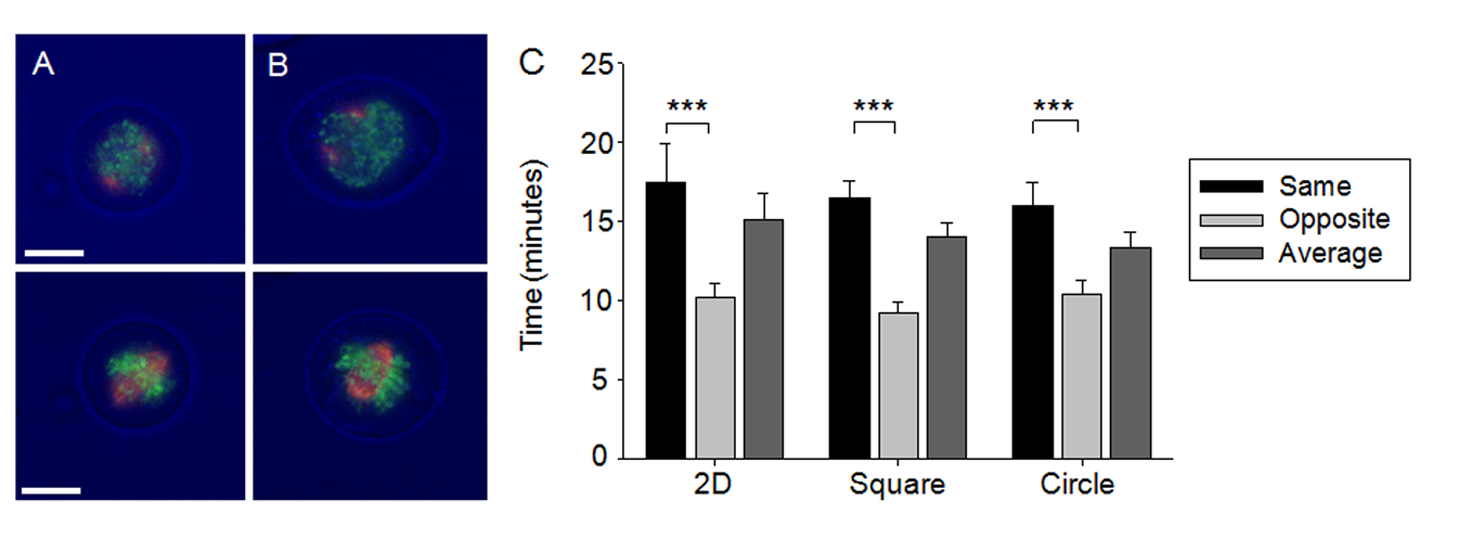

Supplement: Figure S3 — Cell shape did not impact on centrosome separation and spindle formation. (A–B) HeLa cells (RFP-tubulin/GFP-H2B) were synchronized and cultured on the different cell culture platforms and assessed for the position of the centrosomes at NEBD and subsequent spindle formation. Cells were imaged for DNA (green), tubulin (red) and cell outline (transmission, blue); bars: 10 µm. Cells initiated the separation of their centrosomes either (A) during prophase, resulting in centrosomes orthogonal at NEBD or (B) during prometaphase, resulting in centrosomes at the same side of the nuclear envelope at NEBD. (C) Cells with the centrosomes positioned on opposite sides of the nuclear envelope at NEBD were quicker at forming the spindle, regardless of the substrate upon which the cells were cultured. Key: *** p<0.001. (TIF) [file pone.0066918.s003.tif]
